# Modularity without convergence: integrated evolution of the salamander autopodium

**DOI:** 10.1098/rsos.261083

**Published:** 2026-10-21

**Authors:** Giacomo Rosa, Emma Centomo, Andrea Costa, Elisa Damonte, Julien Renet, Antonio Romano, Luca Roner, Sebastiano Salvidio, Sara Ferrando

**Affiliations:** ^1^ University of Genova, Department of Earth, Environment and Life Sciences (DISTAV), Corso Europa 26, I-16132 Genova, Italy; ^2^ National Biodiversity Future Center (NBFC), Piazza Marina 61, I- 90133 Palermo (PA), Italy; ^3^ Research and Museum Collection Office, Conservation Biology Unit, MUSE - Science Museum, Corso del Lavoro e della Scienza 3, 38122 Trento, Italy; ^4^ Fauna studium, Scientific Consulting, 25 Avenue du Jabron, 04200 Sisteron, France; ^5^ Italian National Research Council (CNR), Institute of Bioeconomy – Biology, Agriculture and Food Sciences Department (IBE), Via dei Taurini, 19, 00185 Roma, Italy; ^6^ Departamento de Biología de Organismos y Sistemas, Unidad de Ecología, Universidad de Oviedo, C/ Valentín Andŕes Alvarez s/n, 33006, Oviedo, Spain

**Keywords:** Caudata, landmarks, phylogeny, ecology, foot, modules

## Abstract

Morphological modularity is known to favour evolutionary diversification by enabling different body regions to vary in response to functional and ecological demands. Despite their remarkable diversity in limb morphology and ecology, amphibians—and salamanders in particular—remain significantly under-represented in empirical tests of modularity. Using geometric morphometrics and phylogenetic comparative methods, we investigate patterns of shape variation, integration and modularity in the salamander autopodium. We first validated a landmark-based method to outline foot shape, demonstrating that image orientation does not affect shape analyses and that species can be reliably distinguished. We then applied this approach to a larger comparative dataset encompassing 149 salamander taxa across major lineages and ecological strategies. Our findings demonstrate that the salamander autopodium is functionally modular: the distal region (digits and interdigital region) shows more evolutionary lability than the proximal pes, while remaining integrated within a coordinated evolutionary system. Furthermore, the combinations of allometric, ecological and life-history factors account for a small but significant percentage of shape variation, with a low evolutionary signal in foot shape. Our results suggest that modular organization of the salamander foot may allow for morphological variation while still being limited by a common evolutionary and developmental history. By focusing on an understudied group and morphological system, this study provides new insight on how modularity influences limb evolution in tetrapods.

## 1. Introduction

The interaction between functional requirements, ecological pressures and developmental constraints jointly shapes the evolution of complex morphological structures [1]. Evolutionary change can be significantly influenced by the way these forces are arranged within an organism [2]. The division of anatomical structures into semi-independent modules can promote adaptive diversification while maintaining functional coherence, whereas strong integration can channel variation and limit evolutionary responses [3,4]. However, modular organization does not necessarily imply complete evolutionary independence among anatomical regions, as functional and developmental interactions may maintain coordinated evolutionary responses across modules.

Limbs provide an effective model for testing how integration and modularity influence morphological evolution in vertebrates. Although different limb regions are often subject to different selective pressures related to substrate use, habitat and locomotor mode, limb elements must function as coordinated units during locomotion. Previous studies showed that proximal and distal limb elements may differ in their degree of integration, reflecting both shared developmental processes and specific functional demands [4,5]. Given its direct interaction with the environment and the strong biomechanical and behavioural constraints, the vertebrate distal limb (autopod) is frequently implicated in ecological and evolutionary diversifications [6,7].

While retaining a relatively conserved tetrapod limb bauplan (i.e. the generalized limb organization), salamanders (Caudata) exhibit remarkable ecological and ethological diversities, encompassing a wide range of lifestyles. Salamander feet play a key role in propulsion, adhesion and stability across different substrates [8–10]. Consequently, even subtle variations in digit length, spacing and interdigital morphology can have disproportionate functional consequences [10–13]. Therefore, the salamander autopod represents an ideal system for testing the relationship between patterns of integration, anatomical modularity and ecological diversification, since it is both developmentally well characterized and evolutionarily labile [6,14,15]. This hypothesis is further supported by differences in skeletal organization within salamander autopod, whose proximal elements (i.e. carpals and tarsals) are reduced or remain partially cartilaginous, while distal elements are consistently ossified and structurally conserved [16]. Therefore, proximal and distal regions may respond differently to developmental and functional constraints, potentially promoting their semi-independent evolution.

Despite extensive research on salamander limb development and locomotion [14,17,18], comparative studies explicitly addressing the modular structure of the autopod and its evolutionary implications are still lacking. Previous research focused either on limb proportions, discrete traits (e.g. terrestriality and pentadactyly or relative limb length) or salamander foot shape and their relationship with ecology [6,19–21], demonstrating significant associations between habitat use and autopod morphology. However, these studies did not explicitly investigate whether different regions of the salamander autopod exhibit semi-independent evolutionary dynamics.

We thus combined geometric morphometrics and modularity analyses to investigate the organization of foot shape across the amphibian order of Caudata, analysing autopod morphology in more than 140 species representing nearly all extant salamander genera. We tested if the salamander autopodium is functionally modular, with the distal portion (fingers and interdigital regions) being more evolutionarily variable than the proximal portion (pes). This hypothesis is grounded in functional and ecological considerations: digits are directly involved in substrate interaction, locomotion and habitat adaptation, whereas the pes primarily contributes to structural support and stability and may therefore be more constrained by overall limb morphology [12,18,22]. We further expect ecological categories to differ in distal autopod configuration, reflecting differences in substrate use and locomotor demands.

Our research is timely and addresses key gaps in the current state of art in the context of modularity studies, a field in which amphibians remain strongly under-represented, accounting for only approximately 4% of studied classes, and where Caudata represents just approximately 2% of investigated orders among living organisms [23]. Likewise, shape-based approaches have been used in a limited fraction of modularity studies (approx. 3%), and limbs themselves remain relatively under-represented as a focal morphological system (approx. 5% [23]). By integrating global shape analyses with modularity tests, our approach links ecological diversification with underlying patterns of morphological integration, providing new insight into how modular organization may facilitate evolutionary diversification in salamander limbs. More broadly, this framework allows us to test whether ecological diversification in salamander feet is associated with repeated convergence towards common morphologies or instead reflects multiple evolutionary solutions emerging within a modular but integrated system.

## 2. Methods

Our analytical framework consisted of two sequential steps. First, we used a reduced dataset for validating our landmark method where we tested whether species could be reliably distinguished based on foot shape and whether image orientation influenced shape analyses. Second, once the method was validated, we applied the same digitization protocol to an extended comparative dataset to investigate patterns of shape variation and modularity across salamanders.

### 2.1. Geometric morphometry based on landmarks and semi-landmarks

We digitized salamander hindfoot morphology considering 5 landmarks, positioned on the tips of each digit, and 36 semi-landmarks, distributed along the pes edge and digits’ base (see figure 1A). Landmarks were placed using the *StereoMorph* package (version 4.0.1 [24]) in the R environment, using the function *digitizeImages* on photographs taken perpendicularly to the foot. We followed this approach to capture the whole variability of salamanders’ autopodium [25]. Landmark configurations were aligned using a generalized Procrustes analysis (GPA) implemented in *gpagen* (*geomorph* package), which removes variation owing to size, translation and rotation, allowing analysis of shape differences only. In taxa with digit reduction, missing digits were represented by positioning landmarks at the corresponding anatomical location along the foot margin, following a homologous configuration approach commonly used in geometric morphometrics of structures with variable elements. To account for digit reduction, we maintained landmark homology by assigning landmarks corresponding to missing distal digit tips to the terminal point of the adjacent retained digit. Thus, in four-toed species, the landmarks corresponding to digits IV and V coincided on the fourth digit tip, whereas in tridactyl species, the landmarks corresponding to digits III–V were placed on the third digit tip (supplementary material, figure S1). This approach preserves landmark correspondence among taxa while reflecting the developmental and anatomical reduction of distal digital elements [26].

**Figure 1.**
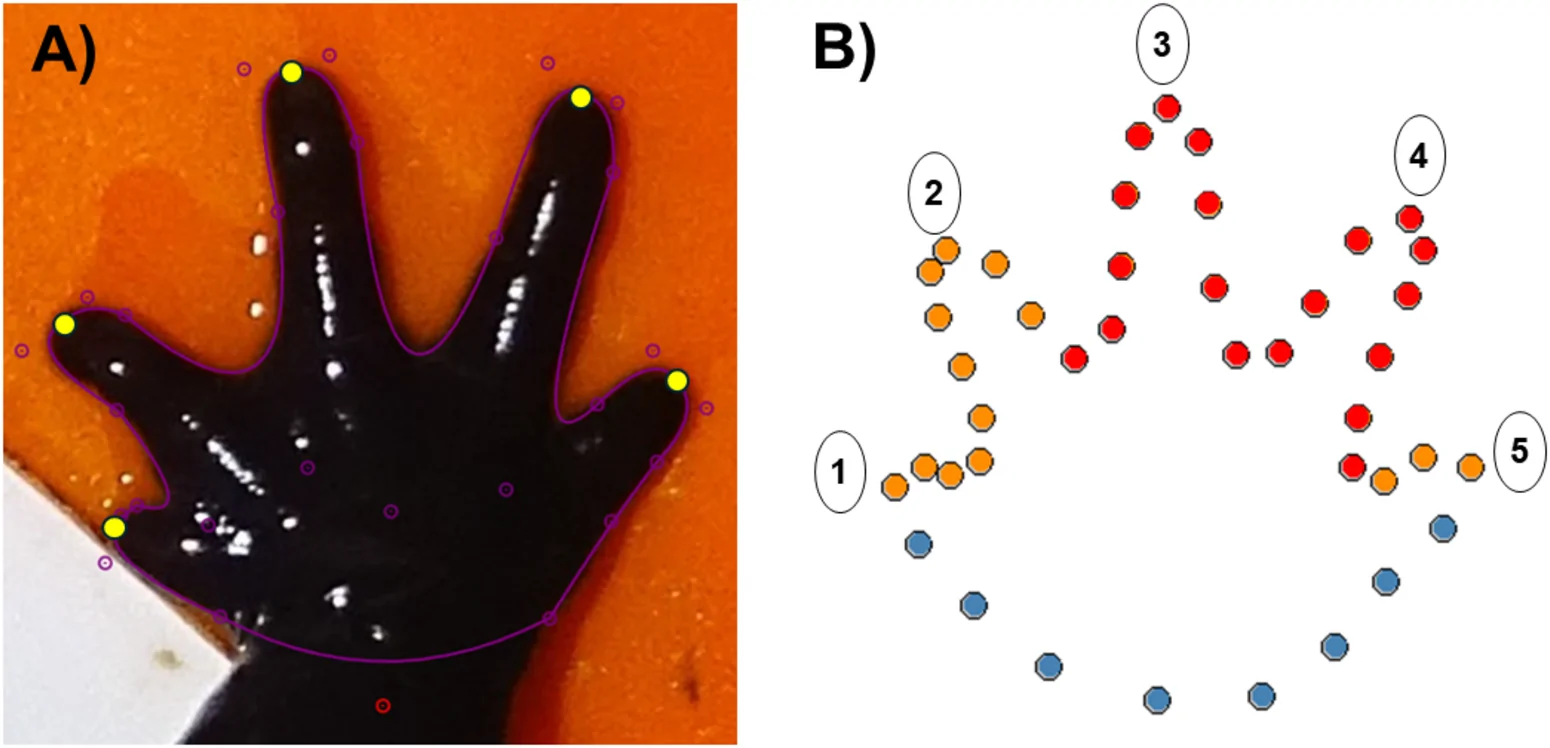
(A) Digitized salamander foot using landmarks (yellow points) and semi-landmarks in *geomorph*. (B) Modules used in the present study: blue points represent proximal pes, while orange ones correspond to the distal region (fingers and webbing) and red points III and IV digits with limiting interdigital regions.

We applied this approach on two different datasets. The first one, used for validating the method, contained seven taxa belonging to the Plethodontidae and Salamandridae families, representing both terrestrial and aquatic strategies. For each taxon, we analysed between 12 and 27 photos, for a total of 135 different individuals (see table 1). Species were chosen based on the availability of multiple high-quality images per taxon and to ensure variation in foot morphology. We used this initial approach to test whether species could be reliably distinguished based on our landmarks’ configuration. In addition, we tested whether image orientation (mirrored left versus right feet) affected identification performance and sexual dimorphic differences among species. To evaluate these effects, we conducted ANOVA-based comparisons on Procrustes-aligned coordinates.

**Table 1.** Species used for testing the methodology with the respective adulthood ecology and number of photos analysed.

species	family	ecology	*n* photos	sex (F – M)	side (L – R)
*Salamandrina perspicillata*^a^	Salamandridae	terrestrial	16	5 – 11	10 – 6
*Salamandra atra*	Salamandridae	terrestrial	20	9 – 10	19 – 0
*Salamandra atra aurorae*	Salamandridae	terrestrial	22	10 – 12	21 – 1
*Euproctus montanus*	Salamandridae	aquatic	27	12 – 15	21 – 6
*Triturus cristatus*	Salamandridae	aquatic	19	11 – 12	19 – 5
*Speleomantes imperialis*	Plethodontidae	terrestrial	19	9 – 11	13 – 7
*Speleomantes strinatii*	Plethodontidae	terrestrial	12	11 – 21	16 – 16

^a^ Four-toed species.

Furthermore, as our dataset contained a four-toed species (*Salamandrina perspicillata*), we repeated the analysis above excluding it to assess the potential influence of digit reduction on our results. The second dataset, used for the broader comparative analysis, consisted of 149 species (with one photo per species, each depicting an adult individual). This sampling represents almost all the 68 genera of Caudata [27], except for genera *Ranodon*, *Phaeognathus* and *Pseudohynobius*, for which usable photographs were not available (see supplementary material, figure S2). Images were retrieved from published literature and openly accessible online sources that permit reuse (i.e. we selected only images explicitly permitting reuse), extracting only landmark-based measurements from the images, without reproducing possible copyrighted material (images included). We distinguished species on the basis of their adulthood ecology (identifying four main groups: aquatic, semiaquatic, terrestrial and arboreal), development (paedomorphic, biphasic and direct) and fertilization mode (external and internal; supplementary material, table S4), reporting also mean snout–vent length (SVL) for each taxon. For dimorphic species, we selectively used the mean SVL corresponding to the specific sex of the specimen included in our study. We derived this classification and measurements from the general information available in standard herpetological literature and recent comparative studies [28,29]. Semiaquatic taxa were defined as species regularly exploiting both aquatic and terrestrial environments for foraging or reproduction. For this dataset, patterns of shape variation and their relationship with size were explored using Procrustes regressions implemented in phylogenetic generalized least squares models (function *procD.pgls*). This allowed us to test the interaction between size (log-SVL) and the categorical variables, directly incorporating the covariance structure among species based on the phylogeny provided by Stewart & Wiens [27]. Before running models, we checked for collinearity between our categorical variables using Pearson's Chi-squared test of independence, where a significant result (*p* < *α* = 0.05, calculated *χ*² > critical *χ*²) indicates collinearity. We then repeated this method on a reduced dataset, excluding taxa with fewer than five digits, to assess the potential influence of digit reduction on our results (i.e. we conducted a sensitivity analysis to check if the number of toes had an effect). We performed model comparison and fit based on Akaike information criterion (AIC) (function *model.comparison*) and ANOVA. We computed mean shapes for each category to visualize shape differences among groups (pairwise comparisons) and visualized shape variation by plotting regression scores against log-SVL. When main effects or interactions were statistically significant, *post hoc* pairwise comparisons between group slope vectors were performed using the pairwise function in the *RRPP* package. Statistical significance for these pairwise distances was evaluated against a null distribution generated through 1000 permutations. We also used principal component analysis (PCA) on Procrustes-aligned coordinates (function *gm.prcomp*) to explore shape variation.

To assess whether category (ecology, development or fertilization mode) led to repeated evolution of similar foot morphologies, we quantified patterns of morphological convergence in a phylogenetic framework. Convergence was evaluated using the function *convnum* implemented in the R package *geomorph* (v. 4.0 [24]). Analyses were performed on phylogenetically informed shape variables obtained from a phylogenetic PCA. We retained the first two principal components, which captured the majority of shape variation and defined a reduced morphospace suitable for convergence assessment. Species scores in this space were used as input, together with the pruned phylogenetic tree. For each categorical factor, we quantified: (i) the number of inferred convergence events (i.e. independent entries into a shared morphospace region) and (ii) the size and structure of the occupied morphospace, represented as a minimum spanning ellipsoid. To test whether the observed number of convergence events exceeded expectations under a null model, we used the function *convnumsig* (package *convevol*), which compares the observed value to a null distribution generated through randomization (100 permutations). Statistical significance was assessed as the proportion of simulated values equal to or exceeding the observed number of events.

All analyses were conducted in R using the packages *StereoMorph*, *geomorph*, *convevol RRPP*, *ape* and *phytools*.

### 2.2. Modularity analysis

We first tested whether ecology, reproductive strategy and phylogeny explained variance when modelling foot as a single integrated structure. After performing all analyses on the full Procrustes-aligned foot configuration, we tested modular organization of salamanders’ autopod using the *phylo.modularity* function in *geomorph*, which explicitly accounts for shared evolutionary history through the phylogenetic covariance structure. We compared three competing models (figure 1B): (i) a non-modular model, where all landmarks were assigned to a single module; (ii) a two main modules model separating distal region (fingers and interdigital regions) from the proximal pes; and (iii) a functional model where landmarks belonging to central digits (III and IV) and adjacent interdigital regions formed a distinct module, reflecting their main role during locomotion.

We used modularity statistic (covariance ratio (CR)) to assess effect strength, where lower CR values indicate stronger modular separation. Statistical support was assessed using 999 random permutations, assessing significance as the proportion of randomized CR values lower than the observed one and comparing alternative hypotheses with the *compare.CR* function.

## 3. Results

The ANOVA-based comparisons on Procrustes-aligned coordinates confirmed that foot shape differed significantly among sampled taxa and that mirroring or view orientation did not introduce systematic bias (supplementary material, figure S3 and table S1). On the other hand, sex showed a significant effect within species (*R*² = 0.03; *F* = 1.6; *p* = 0.01); however, the relative pairwise analyses provide significant results only for *Triturus cristatus* (*F* = 4.38, *Z* = 3.03, *p* = 0.007; supplementary material, table S2), suggesting that sex may play a role in foot shape for certain species. Pairwise interspecific comparison showed significant differences between all species (supplementary material, table S3), except for the two species of genus *Speleomantes* (*F* = 1.93, *Z* = 1.56; *p* = 0.06). The first principal component was mostly influenced by the only species exhibiting digit reduction (*Salamandrina perspicillata*), which was clearly separated from the remaining pentadactyl species (supplementary material, figure S3), highlighting the strong contribution of distal digit morphology to overall foot shape variation. However, the exclusion of *S. perspicillata* from the dataset produced results comparable to the former analysis (supplementary material, table S1 and figure S3), both considering ecology (*R*² = 0.22; *F* = 36.62; *p* = 0.001 versus *R*² = 0.3, *F* = 50.26, *p* = 0.001) and developmental mode (*R*² = 0.15; *F* = 23.73; *p* = 0.001 versus *R*² = 0.7, *F* = 50.26, *p* = 0.001), indicating that foot shape differentiation is not driven only by digit reduction.

### 3.1. Full Procrustes configuration

The first two components of phylogenetically corrected Procrustes PCA explained 51% of total shape variance (supplementary material, figure S4). Because developmental mode was collinear with both ecology and fertilization (*χ*²: 103.15 and 19.33, respectively; *p* < 0.001), we did not build models for phylogenetic MANOVA accounting for more than one categorical variable. SVL showed significant effect in all tested models, explaining a small part of the overall variance (supplementary material, table S5). Ecology and development, both alone and in interaction with SVL (*R*² = 0.05, *Z* = 2.71, *p* = 0.003; *R*² = 0.04, *Z* = 3.03, *p* = 0.001), showed comparable significant results, explaining the largest portion of phylogenetically corrected shape variance. Fertilization mode did not account for significant component variance, neither in interaction with SVL. Sensitivity analysis restricted to five-toed taxa yielded similar results, with ecology and development remaining significant predictors, but with less intensity (supplementary material, table S5). In this subset, the interaction effect between fertilization mode and SVL (*R*² = 0.02, *Z* = 1.86, *p* = 0.03) also showed a significant effect, suggesting that digit reduction or higher number of species may obscure weaker reproductive signals. Model comparison revealed that fertilization mode model has the lowest AIC for both complete and reduced dataset (AIC_All_: −54 066.47, AIC_Reduced_: −48 363.77; supplementary material; table S6), whereas ANOVA showed better fit for ecology (*F*_1__,__4_ = 2.49; *R*² = 0.06, *Z* = 3.35, *p* = 0.001) and development models (*F*_1__,__2_ = 5.17; *R*² = 0.06, *Z* = 5.37, *p* = 0.001), with comparable results (supplementary material, table S7).

Pairwise *RRPP* tests between slope vectors revealed that aquatic species differed strongly from terrestrial (distance—*d* = 0.15, *p* = 0.001; figure 2), while for the other groups did not reveal significant differences (table 2). Interestingly, paedomorphic taxa exhibited significantly different slope trajectories compared to species with both biphasic (*d* = 0.19, *p* = 0.004) and direct development (*d* = 0.21, *p* = 0.01; figure 2 and table 2). Ecological groups showed extensive overlap in the phylogenetic morphospace (supplementary material, figure S4), indicating that the first two phylogenetic principal components capture only a limited portion of ecologically structured variation. Nevertheless, phylogenetic *RRPP* analyses performed on the complete Procrustes shape configuration detected significant differences between aquatic and terrestrial taxa. This apparent discrepancy is normal because ordination plots summarize only a subset of total shape variation, whereas *RRPP* evaluates differences across the full multidimensional shape space.

**Figure 2.**
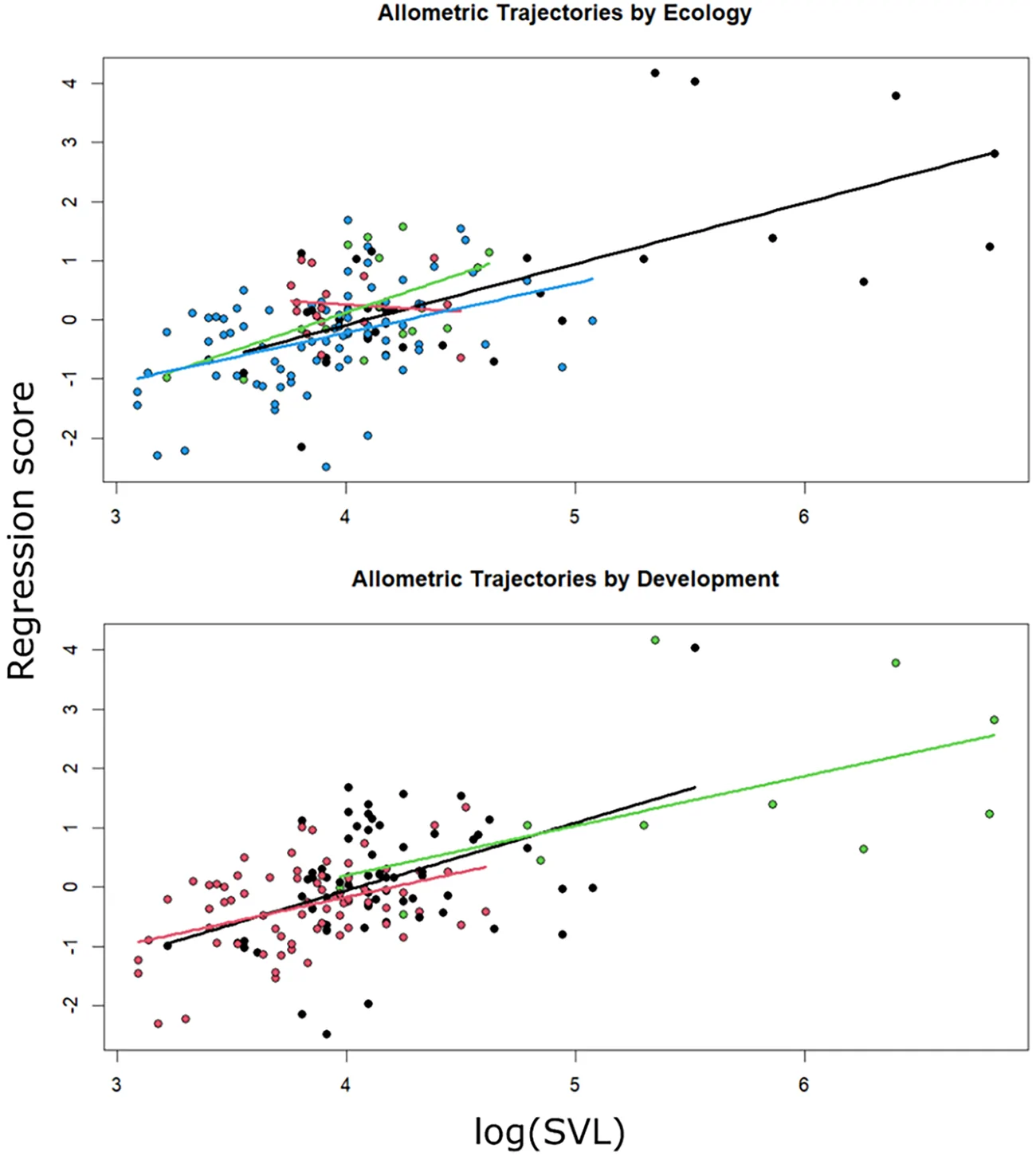
Allometric trajectories of autopodium shape relative to body size grouped by ecology (top panel) and development (bottom panel). Regression scores are plotted against log-transformed snout-vent length (log(SVL)). Points correspond to species and colour to ecological lifestyle (red refers to arboreal, blue to terrestrial, black to aquatic and green to semiaquatic taxa) and development mode (red refers to direct, black to biphasic and green to paedomorphic taxa).

**Table 2.** Pairwise *RRPP* distances between slope vector based on 1000 permutations between selected categorical groups ecology and development.

group	pairwise	*d*	UCL (95%)	*Z*	*p*
ecology	aquatic vs. arboreal	0.21	0.43	−0.91	0.82
	aquatic vs. semiaquatic	0.21	0.22	1.38	0.09
	aquatic vs. terrestrial	0.15	0.11	2.98	**0.001**
	arboreal vs. semiaquatic	0.24	0.46	−0.71	0.76
	arboreal vs. terrestrial	0.15	0.44	−2.22	0.99
	semiaquatic vs. terrestrial	0.15	0.23	0.16	0.44
development	biphasic vs. direct	0.11	0.17	−0.05	0.53
	biphasic vs. paedomorphic	0.19	0.14	2.76	**0.004**
	direct vs. paedomorphic	0.21	0.18	2.28	**0.01**

Bold values indicate statistical significance. *d*, observed distance; UCL (95%), upper confidence limit; *Z*, *Z*-score (effect size); *p*, *p*-value.

Convergence analyses revealed no significant evidence for repeated evolution of similar foot morphologies within ecological and developmental categories (supplementary material, table S8). For ecological groups, the number of inferred convergence events was relatively high (12 events), but not significantly different from expectations under the null model (*p* = 0.67). In addition, the morphospace occupied by ecological categories was large (ellipsoid area approx. 6.17), indicating that species sharing similar ecological strategies are broadly distributed across shape space rather than clustered around a common morphology. This suggests that similar ecological strategies do not lead to occupation of a single adaptive optimum. Instead, taxa sharing comparable ecological conditions appear to retain distinct morphological trajectories within a broadly shared morphospace. Similarly, developmental categories showed a lower number of convergence events (four events), associated with a smaller occupied morphospace (ellipsoid area approx. 1.65), but again without statistical support for convergence (*p* = 0.46).

### 3.2. Module configuration

Phylogenetic modularity analysis revealed significant results for both hypotheses, with the two digits module showing slightly lower CR and stronger effect size (CR = 0.658, *p* = 0.001, *Z* = −4.17) compared to the two main modules model (CR = 0.875, *p* = 0.007, *Z* = −2.50). Bootstrap comparisons indicated that that both modular hypotheses were significantly better supported than a non-modular model (no modules versus proximal–distal model: *Δ* effect size = 2.50, *p* = 0.012; no modules versus central-digits model: *Δ* effect size = 4.17, *p* < 0.001). However, direct comparison between the two modular hypotheses did not reveal significant differences (*Δ* effect size = 0.81, *p* = 0.42), indicating that both partitioning schemes explain covariance patterns similarly well. These results demonstrate that salamanders’ autopodium is modular and could be described by two main modules representing distinct anatomical regions (distal versus proximal region). Both hypotheses identified the distal portion of the foot as the primary source of modular differentiation and evolutionary flexibility, although the partitioning of distal elements is difficult to interpret. Phylogenetic MANOVA on module models showed a high degree of consistency between full autopod shape and anatomical modules. The interaction effects between size (SVL) and biological drivers (ecology and development) remained stable, showing negligible variation of *R*² values and *Z*-scores (see supplementary material, table S9). This indicates that the salamander autopod, although distinguishable into distinct morphological modules, evolves as a highly integrated unit where its different components follow synchronized allometric trajectories.

## 4. Discussion

This study provides a comprehensive phylogenetic morphometric assessment of salamander autopod shape, integrating ecological, developmental and allometric drivers within a modular framework. We show that foot shape diversification is not driven by a single dominant factor but is the result of the interaction between size, ecology and life-history traits.

Importantly, our preliminary analyses validated the robustness of the morphometric approach. The clear identification of the seven species within morphospace and tests on mirrored configurations confirmed that methodological choices did not introduce systematic bias. Although some degree of intraspecific variation related to sex was detected, its overall contribution to interspecific patterns was limited and could not be consistently accounted for across the ‘global’ dataset. Together, these results indicate that the observed macroevolutionary patterns are not artefacts of data acquisition but reflect biologically meaningful variation in autopod morphology. This preliminary analysis anticipated part of the results observed in the phylogenetic corrected dataset, demonstrating the strong influence of ecology (supplementary material, figure S3 and table S1) and the limited effect of digit number (supplementary material, figure S3).

### 4.1. Structural modularity but integrated evolution

Our findings showed how the diversification of salamander foot shape is organized around a clear modular framework, with the distal module (fingers and interdigital region) evolving semi-independently from the proximal pes region.

The ‘two digits’ module, which accounts for the third and fourth digits and the bordering interdigital region (figure 1B), showed slightly stronger modularity signals. However, this result should be interpreted cautiously because these partitions are intrinsically influenced by the same variation they aim to describe. We therefore consider the ‘two modules’ model, which is anatomically defined, as more biologically meaningful, while the ‘two digits’ configuration is better interpreted as identifying regions that contribute to increase overall shape variation. Despite the clear evidence of modularity, the comparative analysis between full shapes and the two modules revealed consistent evolutionary trends. The interaction effects between size (SVL) and biological drivers remained stable across all configurations (full: *Z* = 2.71; two modules: *Z* = 2.54; high loading: *Z* = 2.77), suggesting that while morphological modularity is present and statistically detectable, the autopod evolves as an integrated functional unit (i.e. modularity is morphological, but does not correspond to independent evolutionary process). Indeed, the identified modularity does not lead to divergent evolutionary responses but provides structural flexibility to the entire autopod for responding to selection as a synchronized system. This pattern is consistent with general expectations from evolutionary morphology, where modularity enables localized variation while maintaining overall functional consistency [30]. This apparently contradictory scenario (i.e. modularity without strong evolutionary independence) has been described in other vertebrate systems, where the co-occurrence of developmental and functional factors deeply influences the diversification of individual modules [3,15]. In salamanders, this may reflect shared developmental pathways (e.g. preaxial dominance) and biomechanical constraints that limit the extent to which modules can diverge independently.

### 4.2. Drivers of salamander foot-shape diversification

The evolution of the salamander autopod is not driven by a single dominant factor but by the interplay of multiple drivers, in particular body size, development mode and ecology, that modify ontogenetic pathways. The significant interaction of SVL with ecology and development demonstrates that these factors drive morphological diversity through divergent allometric trajectories. Our results show that semiaquatic taxa follow the steepest allometric slopes (figure 2), suggesting that taxa inhabiting both land and water require morphological adjustments to balance swimming and terrestrial locomotion as body mass increases [6]. By contrast, terrestrial taxa exhibit a shallower allometric trajectory, showing a more stable and biomechanically constrained evolution of foot shape adapted to gravity on land [6]. Aquatic taxa exhibit an intermediate trajectory, occupying distinct regions of the morphospace owing to the larger size range (figure 2). This pattern is linked to the strong co-occurrence of adulthood ecology and developmental mode (figure 2). Indeed, the largest aquatic taxa are almost exclusively paedomorphic. The retention of larval characteristic in adulthood allows these taxa to avoid structural changes imposed by terrestriality, resulting in greater morphological variation (figure 3) and allowing the evolution of giant body size, while keeping aquatic allometric trajectories [17,31]. On the other hand, arboreal taxa exhibit a flat allometric trajectory, indicating that arboreal salamanders maintain the same foot shape across their entire size range. This lack of allometric scaling suggests that foot morphology is optimized early in ontogeny, reflecting particular biomechanical requirements for adhesion and stability on vertical or irregular substrates [32,33]. Also, in some species of bolitoglossine, skin morphology and webbing extension are linked to substrate use [14,34]. In the same group, the presence of interdigital webbing is believed to result from paedomorphic developmental changes (i.e. retention of a juvenile foot morphology), leading to interrelated modifications of distal characteristics, facilitating the recurrent evolution of analogous foot structures despite a lack of significant or uniform selective benefits [14]. The lack of allometry might also reflect a lack of selective pressure on foot shape related to climbing or clinging performance [12].

**Figure 3.**
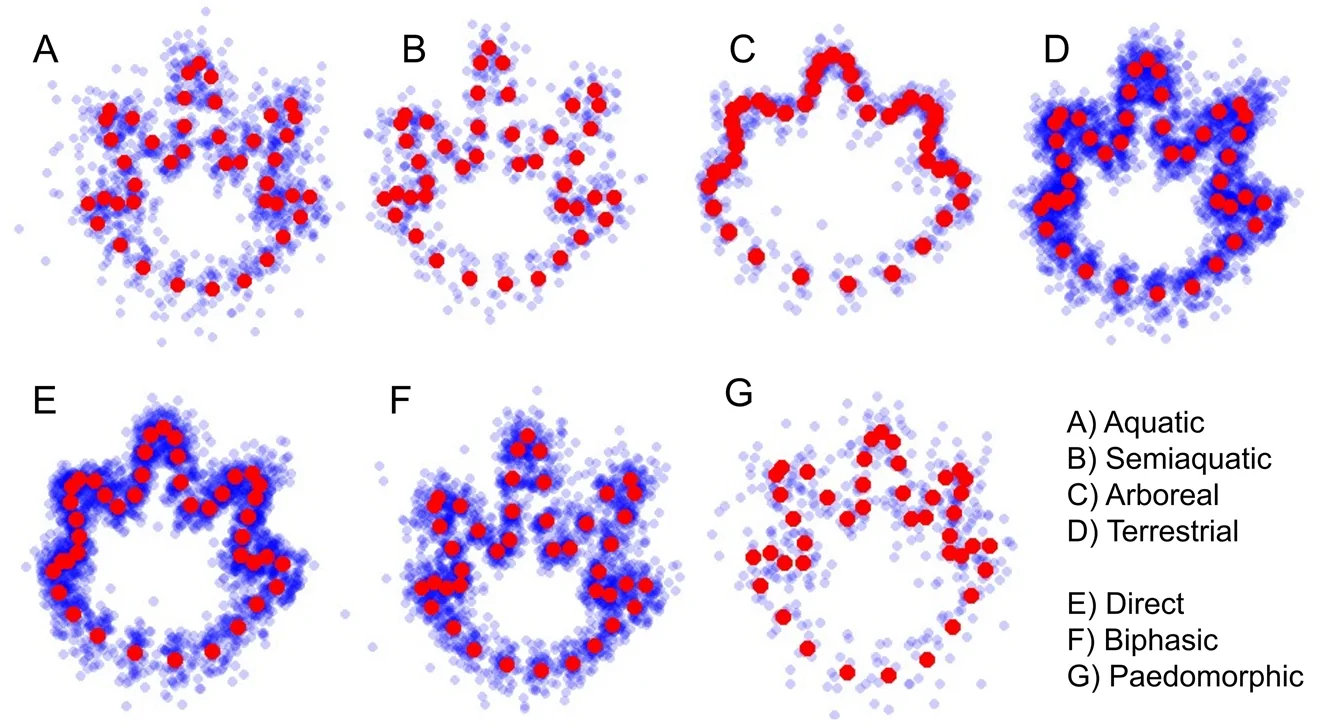
Distribution of landmarks and semi-landmarks of species (blue points) compared to the mean shape (red points) of groups belonging to a category where significant effects were detected (ecology and development).

The significant effect size for development (*Z* = 5.14) was the highest among the tested models, highlighting life-cycle complexity as a primary driver of limb form. This trend may suggest that life-history strategies define the developmental boundaries within which ecological adaptation occurs. Shape variation is highly influenced by the interaction of ossification patterns and developmental timing (e.g. paedomorphosis versus direct development [11,35]). Adult autopodium can be altered by profound shift depending on developmental mode. In paedomorphic taxa, the retention of larval traits into adulthood often results in developmental truncation, resulting in short and poorly ossified digits [11]. Conversely, direct-developing species bypass the aquatic larval stage, avoiding limb modifications owing to aquatic locomotion, and accelerating diversification of foot shapes [6,36]. These results align with previous macroevolutionary analyses in salamanders, showing that shifts in life-cycle complexity alter morphological disparity and evolutionary rates [6,11,36]. Therefore, permanent transitions to single-phase cycles, either aquatic paedomorphs or terrestrial direct developers, seem to lead to higher phenotypic diversification and morphological reorganization.

Importantly, although ecology and development show significant effects, each explains only a small fraction of total variance. This indicates that salamander foot morphology is shaped by multiple weak but interacting drivers, rather than strong deterministic factors.

Results of sensitivity analysis were highly similar compared to the complete dataset, with some minor differences regarding effect size and its significance (see supplementary material, table S5). The slight difference may support the idea that digit number evolution may follow partially independent trajectories relative to overall foot shape [6,11,14]. Since the exclusion of taxa with reduced digit numbers did not produce significant differences, both in the validation and broader dataset, the observed ecological patterns are unlikely to be driven solely by digit reduction. However, repeated evolutionary modifications of distal digital elements, including digit loss in some terrestrial lineages, may contribute to the localized shape changes observed among ecological groups.

### 4.3. Ecological adaptation without strict convergence

Despite the detectable influence of ecological lifestyle (supplementary material, figure S5), species occupying similar ecological niches often remained morphologically distinct and occupied different regions of morphospace, without converging towards a single optimal foot morphology. Rather than evolving towards a single adaptive solution, salamander lineages appear to achieve similar ecological outcomes through alternative morphological configurations, probably reflecting the combined influence of developmental constraints, phylogenetic history and lineage-specific evolutionary trajectories. Visual inspection of the phylogenetic morphospace revealed wide overlap among ecological categories (supplementary material, figure S4), indicating that ecological effects account for only a limited component of total foot shape variation once phylogenetic relationships are considered. Nevertheless, ecological differentiation was not uniformly distributed across groups. Arboreal taxa exhibited the most consistent displacement within morphospace and the most pronounced modifications of distal landmarks (figure 3; supplementary material, figure S4). By contrast, differences between aquatic and terrestrial taxa were more localized and involved a smaller subset of distal structures. This contradiction between morphospace overlap and significant shape differences reflects the fact that ecological variation is concentrated in specific anatomical regions rather than across the entire autopodium. However, some consistent ecological trends emerge, supporting the functional relevance of distal modularity (figure 3). Aquatic and semiaquatic species tend to exhibit elongated and slender digits with reduced palmar expansion, traits associated with stabilization and grasping rather than propulsion, which is primarily driven by the tail [18]. By contrast, terrestrial and arboreal taxa show more robust distal elements and increased webbing, probably enhancing propulsion, substrate contact, climbing and grip [10,22,24]. This pattern is consistent with the modular organization detected in the salamander foot, where variation is concentrated in the distal elements while the proximal region remains comparatively conserved. Our results support the idea that ecological variation appears to be concentrated within distal digital elements, which are crucial for force transmission and grip. Biomechanical and kinematic studies have already demonstrated that the hind limbs of urodeles are crucial in acceleration and propulsion [18,22]. Given that the fourth digit of the foot is usually the longest and most robust, it plays a pivotal role during terrestrial locomotion. This interpretation is consistent with general biomechanical principles that demonstrate that distal limbs in Caudata are crucial for environmental interactions, mediating grip, propulsion and stability [37,38]. Additionally, recent research showed how functional demands and developmental constraints coexist in salamander limbs, allowing localized evolutionary flexibility within a globally integrated structure [15].

### 4.4. A mosaic of evolutionary forces

Overall, our study shows that salamander foot shape is the result of a combination between modularity, integration and multiple ecological and developmental drivers. Although the autopod is structurally modular, its components follow coordinated evolutionary trajectories, indicating persistent functional integration across the limb.

Ecological and developmental factors appear to shape salamander foot diversification primarily through shifts in allometric trajectories rather than through discrete morphological convergence.

This combination of modular organization and functional integration can provide evolutionary flexibility while maintaining functional integrity. Rather than constraining salamander feet towards a single adaptive morphology, modular organization may facilitate the exploration of multiple ecological solutions within a globally integrated locomotor system. More broadly, these findings provide a broader framework for understanding how developmental constraints, integration and ecological pressures interact during tetrapod limb evolution.

## Supplementary Material



## Data Availability

The datasets and R scripts supporting the conclusions of this article are available at https://figshare.com/s/627573c0504bdd43ae81 and are fully cited in the supplementary material [39].

## References

[1] Lauder GV . 1981 Form and function: structural analysis in evolutionary morphology. Paleobiology 7, 430–442. (doi:10.1017/S0094837300025495)

[2] Heylighen F . 1999 The growth of structural and functional complexity during evolution. Evol. Complex. 8, 17–44.

[3] Rothier PS , Simon MN, Marroig G, Herrel A, Kohlsdorf T. 2022 Development and function explain the modular evolution of phalanges in gecko lizards. Proc. R. Soc. B 289, 20212300. (doi:10.1098/rspb.2021.2300) PMC8753168 35016544

[4] Spear JK . 2025 Integration, modularity, and homoplasy in the forelimbs of apes. Evol. Biol. 52, 85–106. (doi:10.1007/s11692-025-09648-9)

[5] Orkney A , Boerma DB, Hedrick BP. 2025 Evolutionary integration of forelimb and hindlimb proportions within the bat wing membrane inhibits ecological adaptation. Nat. Ecol. Evol. 9, 111–123. (doi:10.1038/s41559-024-02572-9) 39487310

[6] Ledbetter NM , Bonett RM. 2019 Terrestriality constrains salamander limb diversification: implications for the evolution of pentadactyly. J. Evol. Biol. 32, 642–652. (doi:10.1111/jeb.13444) 30891861

[7] Fitzpatrick BM . 2025 Trade-offs between aquatic and terrestrial locomotion and functional parallelism in *Desmognathus* salamanders. PeerJ 13, e20111. (doi:10.7717/peerj.20111) PMC12510250 41081098

[8] Huie JM , Park G, Kawano SM. 2025 Salamander ecomorphology reveals a unique suite of climbing adaptations. Proc. R. Soc. B 292, 20251295. (doi:10.1098/rspb.2025.1295) PMC12380490 40858261

[9] Adams DC , Nistri A. 2010 Ontogenetic convergence and evolution of foot morphology in European cave salamanders (Family: Plethodontidae). BMC Evol. Biol. 10, 216. (doi:10.1186/1471-2148-10-216) PMC2927916 20637087

[10] Brown CE , Goldenberg WP, Hinds OM, O'Donnell MK, Staub NL. 2025 Vascular and osteological morphology of expanded digit tips suggests specialization in the wandering salamander (*Aneides vagrans*). J. Morphol. 286, e70026. (doi:10.1002/jmor.70026) PMC11711880 39780375

[11] Wiens JJ , Hoverman JT. 2008 Digit reduction, body size, and paedomorphosis in salamanders. Evol. Dev. 10, 449–463. (doi:10.1111/j.1525-142X.2008.00256.x) 18638322

[12] Baken EK , O'Donnell MK. 2021 Clinging ability is related to particular aspects of foot morphology in salamanders. Ecol. Evol. 11, 11 000–11 008. (doi:10.1002/ece3.7888) PMC8366850 34429897

[13] Adams DC , Korneisel D, Young M, Nistri A. 2017 Natural history constrains the macroevolution of foot morphology in European plethodontid salamanders. Am. Nat. 190, 292–297. (doi:10.1086/69247) 28731800

[14] Jaekel M , Wake DB. 2007 Developmental processes underlying the evolution of a derived foot morphology in salamanders. Proc. Natl Acad. Sci. USA 104, 20 437–20 442. (doi:10.1073/pnas.0710216105) PMC2154449 18077320

[15] Riquelme-Guzmán C , Sandoval-Guzmán T. 2024 The salamander limb: a perfect model to understand imperfect integration during skeletal regeneration. Biol. Open 13, bio060152. (doi:10.1242/bio.060152) PMC10868587 38319134

[16] Fröbisch NB . 2008 Ossification patterns in the tetrapod limb–conservation and divergence from morphogenetic events. Biol. Rev. 83, 571–600. (doi:10.1111/j.1469-185X.2008.00055.x) 18947337

[17] Vega CM , Ashley-Ross MA. 2020 Tiger salamanders (*Ambystoma tigrinum*) increase foot contact surface area on challenging substrates during terrestrial locomotion. Integr. Org. Biol. 2, obaa029. (doi:10.1093/iob/obaa029) PMC7794020 33791568

[18] Kawano SM , Blob RW. 2013 Propulsive forces of mudskipper fins and salamander limbs during terrestrial locomotion: implications for the invasion of land. Integr. Comp. Biol. 53, 283–294. (doi:10.1093/icb/ict051) 23667046

[19] Rosa G , Costa A, Bozzo M, Salvidio S, Ferrando S. In press. A tale of appendages: investigating limb and tail variation in salamanders. Integr. Zool. (doi:10.1111/1749-4877.70050) 41538309

[20] Baken EK , Adams DC. 2019 Macroevolution of arboreality in salamanders. Ecol. Evol. 9, 7005–7016. (doi:10.1002/ece3.5267) PMC6662381 31380029

[21] Ponssa ML , Fratani J, Barrionuevo JS. 2022 Phalanx morphology in salamanders: a reflection of microhabitat use, life cycle or evolutionary constraints? Zoology 154, 126040. (doi:10.1016/j.zool.2022.126040) 35970064

[22] Pierce SE , Lamas LP, Pelligand L, Schilling N, Hutchinson JR. 2020 Patterns of limb and epaxial muscle activity during walking in the fire salamander, *Salamandra salamandra*. Integr. Org. Biol. 2, obaa015. (doi:10.1093/iob/obaa015) PMC7671131 33791558

[23] Esteve-Altava B . 2017 In search of morphological modules: a systematic review. Biol. Rev. 92, 1332–1347. (doi:10.1111/brv.12284) 27245113

[24] Baken EK , Collyer ML, Kaliontzopoulou A, Adams DC. 2021 *geomorph* v4.0 and gmShiny: enhanced analytics and a new graphical interface for a comprehensive morphometric experience. Methods Ecol. Evol. 12, 2355–2363. (doi:10.1111/2041-210X.13723)

[25] Shubin NH , Daeschler EB, Jenkins Jr FA. 2006 The pectoral fin of *Tiktaalik roseae* and the origin of the tetrapod limb. Nature 440, 764–771. (doi:10.1038/nature04637) 16598250

[26] Zelditch ML , Swiderski DL, Sheets HD. 2012 Geometric morphometrics for biologists: a primer, 2nd edn. London, UK: Academic Press.

[27] Stewart AA , Wiens JJ. 2025 A time-calibrated salamander phylogeny including 765 species and 503 genes. Mol. Phylogenet. Evol. 204, 108272. (doi:10.1016/j.ympev.2024.108272) 39681150

[28] Huang N , Sun X, Song Y, Yuan Z, Zhou W. 2023 Amphibian traits database: a global database on morphological traits of amphibians. Global Ecol. Biogeogr. 32, 633–641. (doi:10.1111/geb.13656)

[29] Griffiths RA , Teunis B. 1996 Newts and salamanders of Europe. London, UK: T & AD Poyser. https://library.wur.nl/WebQuery/titel/936963.

[30] Esteve-Altava B . 2017 Challenges in identifying and interpreting organizational modules in morphology. J. Morphol. 278, 960–974. (doi:10.1002/jmor.20690) 28466514

[31] Delvolvé I , Bem T, Cabelguen J-M. 1997 Epaxial and limb muscle activity during swimming and terrestrial stepping in the adult newt, *Pleurodeles waltl*. J. Neurophysiol. 78, 638–650. (doi:10.1152/jn.1997.78.2.638) 9307101

[32] Labonte D , Clemente CJ, Dittrich A, Kuo C-Y, Crosby AJ, Irschick DJ, Federle W. 2016 Extreme positive allometry of animal adhesive pads and the size limits of adhesion-based climbing. Proc. Natl Acad. Sci. USA 113, 1297–1302. (doi:10.1073/pnas.1519459113) PMC4747726 26787862

[33] Labonte D , Federle W. 2015 Scaling and biomechanics of surface attachment in climbing animals. Phil. Trans. R. Soc. B 370, 20140027. (doi:10.1098/rstb.2014.0027) PMC4275900 25533088

[34] Green DM , Alberch P. 1981 Interdigital webbing and skin morphology in the neotropical salamander genus *Bolitoglossa* (Amphibia; Plethodontidae). J. Morphol. 170, 273–282. (doi:10.1002/jmor.1051700302) 30119588

[35] Jia J , Anderson JS, Jiang J-P, Wu W, Shubin NH, Gao K-Q. 2022 Ossification patterns of the carpus and tarsus in salamanders and impacts of preaxial dominance on the fin-to-limb transition. Sci. Adv. 8, eabq7669. (doi:10.1126/sciadv.abq7669) PMC9565805 36240271

[36] Bonett RM , Blair AL. 2017 Evidence for complex life cycle constraints on salamander body form diversification. Proc. Natl Acad. Sci. USA 114, 9936–9941. (doi:10.1073/pnas.1703877114) PMC5604006 28851828

[37] Russell A , Bels V. 2001 Biomechanics and kinematics of limb-based locomotion in lizards: review, synthesis and prospectus. Comp. Biochem. Physiol. A Mol. Integr. Physiol. 131, 89–112. (doi:10.1016/S1095-6433(01)00469-X) 11733169

[38] Molnar JL . 2021 Variation in articular cartilage thickness among extant salamanders and implications for limb function in stem tetrapods. Front. Ecol. Evol. 9, 671006. (doi:10.3389/fevo.2021.671006)

[39] Rosa G , Centomo E, Costa A, Damonte E, Renet J, Romano A, Roner L, Salvidio S, Ferrando S. 2026 Supplementary material from: Modularity without convergence: integrated evolution of the salamander autopodium. Figshare. (doi:10.6084/m9.figshare.c.8693409)

