# Supplementary material for: Modularity without convergence: integrated evolution of the salamander autopodium
Source: R Soc Open Sci. 2026 Oct 21;13(10):261083. doi: 10.1098/rsos.261083 (PMC13682868; doi:10.1098/rsos.261083)
Supplement: Supplementary file 1 [file rsos.261083.zip › rsos.261083/rsos261083_si_001.docx]

**Modularity without convergence: integrated evolution of the salamander autopodium**

**Figure S1** Landmark assignment in species (in this case S. perspicillata) with reduced digit number.

**Figure S2** Phylogenetic tree of the 149 species included in the study. Colors represent salamander families: red (Hynobiidae), orange (Amphiumidae), green (Cryptobranchidae), blue (Ambystomatidae), pink (Rhyacotritonidae), olive (Salamandridae), purple (Plethodontidae), and brown (Proteidae).

**Figure S3** Scatterplot of the geometric morphometric PCA conducted on salamander foot shape. Each point represents an individual, and colors correspond to adulthood ecologies, with (A) and without (B) *S. perspicillata*, and species (C; EuMon: *Euproctus montanus*, Salatr: *Salamandra atra*, Salaur: *S. a. aurorae*, Salper: *Salamandrina perspicillata*, Speimp: *Speleomantes imperialis*, Spestr: *Speleomantes strinatii*, Tricri: *Triturus cristatus*). Ellipses represent the 95% confidence region for each species, highlighting differences in foot morphology.

**Table S1** Results of MANOVA on full autopod shape, using a GLS approach. The table reports degrees of freedom (Df), sums of squares (SS), coefficient of determination (R²), F-statistics, and permutation-based p-values.

|  |  | **All seven species** | | | | | **Five digits only** | | | | |
| --- | --- | --- | --- | --- | --- | --- | --- | --- | --- | --- | --- |
| **Model** | **Effect** | **Df** | **SS** | **R²** | **F** | **p** | **Df** | **SS** | **R²** | **F** | **p** |
| Species | Species | 6 | 2.37 | 0.61 | 33.02 | **0.001** | 5 | 0.94 | 0.43 | 16.92 | **0.001** |
| Ecology | Ecology | 1 | 0.84 | 0.22 | 36.62 | **0.001** | 1 | 0.66 | 0.3 | 50.26 | **0.001** |
| Development | Development | 1 | 0.6 | 0.15 | 23.73 | **0.001** | 1 | 0.7 | 0.3 | 50.26 | **0.001** |
| Species×Sex | Species | 6 | 2.37 | 0.61 | 33.02 | **0.001** | **5** | 0.94 | 0.43 | 17.68 | **0.001** |
|  | Sex | 1 | 0.03 | 0.007 | 1.1 | 0.33 | 1 | 0.03 | 0.01 | 1.2 | 0.28 |
|  | Species:Sex | 5 | 0.11 | 0.03 | 1.6 | **0.01** | 5 | 0.10 | 0.05 | 1.9 | **0.003** |
| Species×Side | Species | 6 | 2.37 | 0.61 | 33.02 | **0.001** | 5 | 0.94 | 0.43 | 16.62 | **0.001** |
|  | Side | 1 | 0.007 | 0.002 | 0.6 | 0.77 | 1 | 0.007 | 0.003 | 0.58 | 0.79 |
|  | Species:Side | 5 | 0.04 | 0.009 | 0.6 | 0.95 | 4 | 0.03 | 0.01 | 0.61 | 0.91 |

**Table S2** Within species comparisons between sexes, based on PCA scores derived from foot shape. Reported values are F statistics, Z scores, and relative P values. All significant differences (P ≤ 0.05) indicate detectable divergence in foot morphology between sexes

| Species | F | Z | p |
| --- | --- | --- | --- |
| *Euproctus montanus* | 1.55 | 0.94 | 0.31 |
| *Salamandra atra* | 1.58 | 1.13 | 0.31 |
| *Salamandra a. aurorae* | 1.00 | 0.26 | 0.47 |
| *Salamandrina perspicillata* | 0.49 | -0.73 | 0.75 |
| *Speleomantes imperialis* | 1.51 | 1.01 | 0.31 |
| *Speleomantes strinatii* | 1.06 | 0.27 | 0.47 |
| *Triturus cristatus* | 4.38 | 3.03 | **0.007** |

**Table S3** Pairwise comparisons between species based on PCA scores derived from foot shape. Reported values are F statistics, Z scores, and relative P values. All significant differences (P ≤ 0.05) indicate detectable divergence in foot morphology between the corresponding species and adulthood ecology. Pedices “all” indicate analysis including all taxa, while “red” after removing *S. perspicillata*.

| Species1 | Species2 | F | Z | P |
| --- | --- | --- | --- | --- |
| EuMon | Salatr | 14.170 | 5.138 | **0.001** |
| EuMon | Salaur | 10.738 | 4.237 | **0.001** |
| EuMon | Salper | 62.822 | 4.408 | **0.001** |
| EuMon | Speimp | 25.337 | 6.230 | **0.001** |
| EuMon | Spestr | 18.429 | 5.334 | **0.001** |
| EuMon | Tricri | 4.401 | 3.651 | **0.001** |
| Salatr | Salaur | 2.581 | 2.116 | **0.019** |
| Salatr | Salper | 79.918 | 4.763 | **0.001** |
| Salatr | Speimp | 14.511 | 5.361 | **0.001** |
| Salatr | Spestr | 12.171 | 4.685 | **0.001** |
| Salatr | Tricri | 24.917 | 4.290 | **0.001** |
| Salaur | Salper | 69.827 | 5.033 | **0.001** |
| Salaur | Speimp | 14.285 | 5.077 | **0.001** |
| Salaur | Spestr | 12.730 | 4.555 | **0.001** |
| Salaur | Tricri | 21.206 | 4.483 | **0.001** |
| Salper | Speimp | 60.044 | 3.876 | **0.001** |
| Salper | Spestr | 58.415 | 4.921 | **0.001** |
| Salper | Tricri | 71.147 | 3.585 | **0.001** |
| Speimp | Spestr | 1.927 | 1.563 | 0.059 |
| Speimp | Tricri | 38.869 | 3.907 | **0.001** |
| Spestr | Tricri | 28.675 | 3.887 | **0.001** |
| Aquatic | Terrestrial_all_ | 33.017 | 12.278 | **0.001** |
| Aquatic | Terrestrial_red_ | 36.63 | 5.6 | **0.001** |

**Table S4** Number of species distinguished on the basis of their adulthood ecology, fertilization and development mode.

|  | **Direct** | | **Biphasic** | | **Paedomorphic** | | **Total** |
| --- | --- | --- | --- | --- | --- | --- | --- |
|  | **External** | **Internal** | **External** | **Internal** | **External** | **Internal** |  |
| **Aquatic** | 0 | 0 | 1 | 21 | 3 | 8 | 33 |
| **Arboreal** | 0 | 17 | 0 | 0 | 0 | 0 | 17 |
| **Semiaquatic** | 0 | 0 | 4 | 13 | 0 | 0 | 17 |
| **Terrestrial** | 0 | 57 | 10 | 18 | 0 | 0 | 84 |
| **Total** | 0 | 74 | 15 | 52 | 3 | 8 | 151 |

**Table S5** Results of phylogenetic MANOVA on full autopod shape, using a GLS approach incorporating the phylogenetic covariance matrix. The table reports degrees of freedom (Df), Sums of Squares (SS), R-squaered (R²), F-statistics (F), and p-values (p).

| **Model** | **Effect** | **All** | | | | | | **Five digits only** | | | | | |
| --- | --- | --- | --- | --- | --- | --- | --- | --- | --- | --- | --- | --- | --- |
|  |  | **Df** | **SS** | **R²** | **F** | **Z** | **p** | **Df** | **SS** | **R²** | **F** | **Z** | **p** |
| Eco×log(SVL) | Eco | 3 | 3.86 | 0.04 | 2.3 | 2.17 | **0.02** | 3 | 3.81 | 0.06 | 2.78 | 2.46 | **0.009** |
|  | SVL | 1 | 1.48 | 0.02 | 2.63 | 2.19 | **0.02** | 1 | 1.25 | 0.02 | 2.74 | 2.19 | **0.02** |
|  | Eco:SVL | 3 | 4.19 | 0.05 | 2.49 | 2.71 | **0.003** | 3 | 2.64 | 0.04 | 1.93 | 2.08 | **0.02** |
| Fert×log(SVL) | Fert | 1 | 1.04 | 0.01 | 1.77 | 1.11 | 0.125 | 1 | 0.84 | 0.01 | 1.78 | 1.15 | 0.13 |
|  | SVL | 1 | 1.98 | 0.02 | 3.39 | 2.63 | **0.001** | 1 | 1.5 | 0.02 | 3.16 | 2.44 | **0.008** |
|  | Fert:SVL | 1 | 0.93 | 0.01 | 1.59 | 2.05 | 0.133 | 1 | 1.21 | 0.02 | 2.55 | 1.86 | **0.03** |
| Develop×log(SVL) | Develop | 2 | 4.57 | 0.05 | 4.13 | 2.46 | **0.008** | 2 | 4.8 | 0.07 | 5.39 | 2.87 | **0.03** |
|  | SVL | 1 | 1.32 | 0.01 | 2.39 | 1.95 | **0.02** | 1 | 1.26 | 0.02 | 2.84 | 2.26 | **0.01** |
|  | Develop:SVL | 2 | 3.77 | 0.04 | 3.41 | 3.03 | **0.001** | 2 | 2.15 | 0.03 | 2.42 | 2.15 | **0.02** |

**Table S6** Model selection following AIC values for the complete and filtered dataset, using “model.comparison()” function. Residual.pc.no. corresponds to residual percentage (or proportional) number of parameters.

| **Dataset** | **Model** | **logLik** | **Residual.pc.no** | **Penalty** | **AIC** |
| --- | --- | --- | --- | --- | --- |
| All | Fert×log(SVL) | 30939.23 | 84 | 7812 | -54066.47 |
|  | Develop×log(SVL) | 31008.25 | 84 | 8148 | -53868.50 |
|  | Eco×log(SVL | 31007.37 | 84 | 8484 | -53530.75 |
| Five digits only | Fert×log(SVL) | 28087.89 | 84 | 7812 | -48363.77 |
|  | Develop×log(SVL) | 28155.04 | 84 | 8148 | -48162.07 |
|  | Eco×log(SVL | 28151.85 | 84 | 8484 | -47819.69 |

**Table S7** Model fit following ANOVA for complete and reduced dataset based on ANOVA. Df: Degrees of Freedom, RSS: Residual Sum of Squares, SS: Sum of Squares, R²: R-squared, F: F-statistic, Z: Z-score, p: p-value.

| **Dataset** | **Model** | **Df** | **RSS** | **SS** | **R²** | **F** | **Z** | **p** |
| --- | --- | --- | --- | --- | --- | --- | --- | --- |
| All | Fert×log(SVL) | 1 | 61.57 | Null | | | | |
|  | Develop×log(SVL) | 2 | 56.91 | 5.72 | 0.06 | 5.17 | 5.37 | **0.001** |
|  | Eco×log(SVL | 4 | 57.42 | 5.59 | 0.06 | 2.49 | 3.35 | **0.001** |
| Five digits only | Fert×log(SVL) | 1 | 61.57 | Null | | | | |
|  | Develop×log(SVL) | 2 | 56.91 | 4.66 | 0.07 | 5.24 | 5.37 | **0.001** |
|  | Eco×log(SVL) | 4 | 57.42 | 4.14 | 0.06 | 2.27 | 3.35 | **0.005** |

**Figure S4** Phylogenetically corrected Procrustes ANOVA. Points correspond to species and colour to ecological lifestyle (red refers to arboreal, blue to terrestrial, black to aquatic and green to semiaquatic taxa) and development mode (red refers to direct, black to biphasic and green to paedomorphic taxa).

**Table S8** Results of convergence analysis. Simulated events are based on 1,000 randomizations under a Brownian motion null model.

| **Category Type** | **Observed Convergence Events** | **Simulated Events (Null Model Mean ± SD)** | **Morphospace Size (Ellipsoid Area)** | **p-value** |
| --- | --- | --- | --- | --- |
| **Ecology** | 12 | 11.3 **±** 5.1 | 6.17 | 0.67 |
| **Development** | 4 | 7.48 **±** 4.86 | 1.65 | 0.75 |

**Table S9** Results of phylogenetic MANOVA on autopod shape, distinguished based on the two modular, models using a GLS approach incorporating the phylogenetic covariance matrix. The table reports degrees of freedom (Df), Sums of Squares (SS), R-squaered (R²), F-statistics (F), and p-values (p).

| **Model** | **Effect** | **Two modules** | | | | | | **High loading** | | | | | |
| --- | --- | --- | --- | --- | --- | --- | --- | --- | --- | --- | --- | --- | --- |
|  |  | **Df** | **SS** | **R²** | **F** | **Z** | **p** | **Df** | **SS** | **R²** | **F** | **Z** | **p** |
| Eco×log(SVL) | Eco | 3 | 3.18 | 0.05 | 2.4 | 2.19 | **0.02** | 3 | 2.91 | 0.05 | 2.42 | 2.23 | **0.02** |
|  | SVL | 1 | 1.146 | 0.02 | 2.6 | 2.13 | **0.02** | 1 | 0.94 | 0.01 | 2.34 | 1.8 | **0.04** |
|  | Eco:SVL | 3 | 3.2 | 0.05 | 2.42 | 2.54 | **0.007** | 3 | 3.18 | 0.05 | 2.66 | 2.77 | **0.004** |
| Fert×log(SVL) | Fert | 1 | 0.8 | 0.01 | 1.73 | 1.05 | 0.145 | 1 | 0.8 | 0.01 | 1.91 | 1.22 | 0.105 |
|  | SVL | 1 | 1.52 | 0.02 | 3.29 | 2.6 | **0.004** | 1 | 1.39 | 0.02 | 3.34 | 2.45 | **0.005** |
|  | Fert:SVL | 1 | 0.59 | 0.008 | 1.29 | 0.75 | 0.22 | 1 | 0.782 | 0.01 | 1.88 | 1.28 | 0.096 |
| Develop×log(SVL) | Develop | 2 | 3.4 | 0.05 | 4.19 | 2.4 | **0.01** | 2 | 3.45 | 0.05 | 4.4 | 2.5 | **0.009** |
|  | SVL | 1 | 1.02 | 0.01 | 2.35 | 1.9 | **0.03** | 1 | 0.83 | 0.01 | 2.12 | 1.59 | 0.057 |
|  | Develop:SVL | 2 | 2.29 | 0.04 | 3.35 | 2.96 | **0.001** | 2 | 3.11 | 0.05 | 3.97 | 3.16 | **0.001** |

**Figure S5** Shape variation between groups of categories where significant effects were detected (Ecology and Development).
